# Altered OXPHOS potential in ascites immune cells is linked to CD4 T-cell exhaustion and a higher risk of spontaneous bacterial peritonitis in cirrhosis

**DOI:** 10.3389/fimmu.2026.1853973

**Published:** 2026-10-06

**Authors:** Hasan Tarik Cosgun, Pavitra Kumar, Ingrid Wei Zhang, Linda Hammerich, Virein Sudheer, Charalampos Pavlidis, Juan Wang, Hannah Schwarz, Frank Tacke, Ali Canbay, Cornelius Engelmann

**Affiliations:** 1 Department of Hepatology & Gastroenterology, Charité – Universitätsmedizin Berlin, Campus Virchow-Klinikum and Campus Charité Mitte, Berlin, Germany; 2 Section of Hepatology and Gastroenterology, Department of Internal Medicine, Knappschaft Kliniken-University Hospital Bochum, Ruhr University Bochum, Bochum, Germany

**Keywords:** cirrhosis, immune cells, immunometabolism, mitochondria, spontaneous bacterial peritonitis

## Abstract

**Background and aims:**

Ascites marks advanced cirrhosis and increases the risk of complications including SBP. Systemic inflammation in liver disease is well characterized, but the immunometabolic properties of the peritoneal compartment remain undefined. This study evaluated the metabolic characteristics of ascites-derived immune cells.

**Method:**

Matched blood and ascites samples from patients with cirrhosis without SBP (n = 21) were analyzed. OXPHOS and glycolysis were assessed using the Seahorse XFe96, lactate concentrations were measured, and immune cell subsets were characterized by spectral flow cytometry.

**Results:**

T cells were the dominant subset in ascites and blood (58.2% vs. 49%, p=0.0822) and CD4^+^ T cells represented the predominant subset (57.3% vs. 64.6%, p=0.026). Ascites-derived MNCs exhibited higher basal and maximal respiration than blood-counterparts (4.4 vs 3.6, p = 0.033 and 20 vs 13.3 pmol O_2_/min/µg protein, p=0.004). Proton leak was increased in ascites (0.7 vs 0.4 pmol O_2_/min/µg protein, p<0.0001). OXPHOS potential was increased in ascites (365.9% vs. 290.9%, p<0.001) while glycolytic potential was reduced (208.0% vs. 286.8%, p < 0.001), corroborated by lower ascitic lactate concentrations (1.9 vs. 3.8 mM/L, p<0.001). Patients with low ascites OXPHOS potential had higher risk of developing SBP (p = 0.016). CD4^+^ T cells in ascites showed elevated PD-1 expression (36.9% vs. 14.7%, p < 0.0001), associated with reduced OXPHOS potential (407.1% vs. 324.6% in PD-1-low vs. PD-1-high cells, p = 0.036).

**Conclusion:**

Peritoneal immune cells exhibit a dissociated phenotype—elevated oxidative activity alongside features of immune exhaustion—reflecting their distinct role in immune regulation within the peritoneal environment.

## Graphical Abstract

**Figure:**
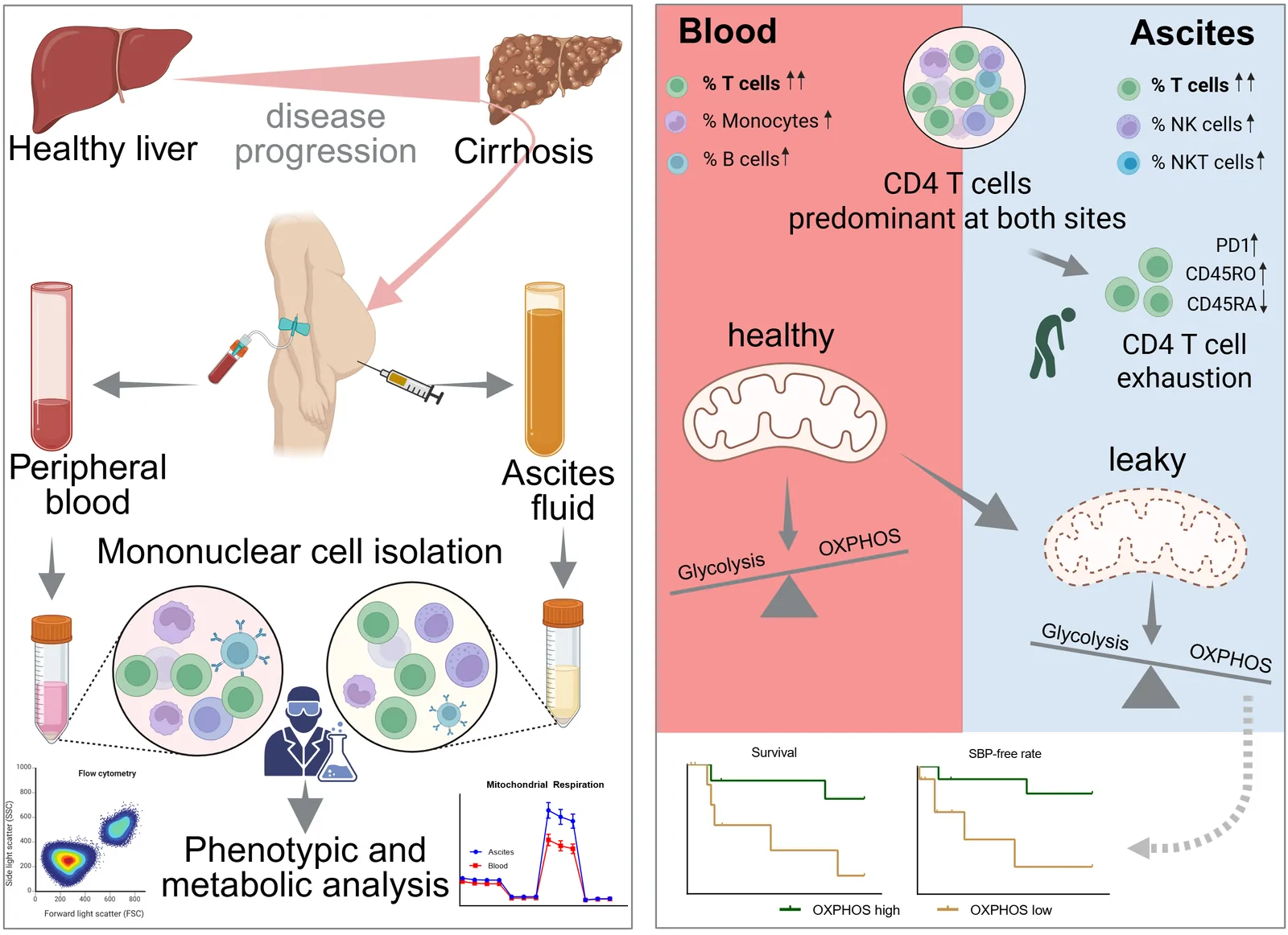

## Highlights

- Peritoneal mononuclear cells in advanced cirrhosis show heightened metabolic activity with increased oxidative phosphorylation, elevated proton leak, and reduced glycolytic potential compared with blood cells.
- Ascites CD4^+^ T cells exhibit marked exhaustion, with higher PD-1 expression and a dominant effector-memory phenotype.
- Low OXPHOS potential in ascites immune cells correlates with a higher one-year risk of spontaneous bacterial peritonitis (SBP).
- Findings reveal distinct immune metabolic dysregulation in the peritoneal compartment that may influence infection susceptibility in cirrhosis.

## 1 Introduction

Liver disease remains a major global health challenge, contributing to approximately two million deaths annually (1). Cirrhosis is a progressive condition associated with significant morbidity and mortality and its decompensation marks a critical turning point in disease progression (2). Among its complications, ascites is the most frequent and one of the earliest clinical indicators of decompensation, often leading to further severe outcomes such as spontaneous bacterial peritonitis (SBP), renal dysfunction, gastrointestinal bleeding, and acute-on-chronic liver failure (ACLF), all contributing to high short-term mortality (3, 4).

As cirrhosis progresses, it is increasingly recognized as a condition marked by profound disturbances in both innate and adaptive immunity. This dysfunction is collectively referred to as cirrhosis-associated immune dysfunction (CAID) and is characterized by a paradoxical coexistence of systemic inflammation and immune suppression (5, 6).

Recent research has linked immune imbalance to changes in immune cell metabolism. Metabolic shifts not only respond to immune activation but also shape immune cell differentiation, function, and fate (7). A defining component of these metabolic shifts is mitochondrial dysfunction, which plays a central role in shaping immune cell bioenergetics and immune behavior. Zhang and colleagues demonstrated structural and functional mitochondrial alterations in circulating leukocytes from patients with acutely decompensated cirrhosis and ACLF, indicating a reprogramming in core metabolic pathways and a need for compensatory mechanisms to maintain energy production (8). Similarly, studies in patients with metabolic dysfunction–associated steatotic liver disease (MASLD) have shown that blood-derived immune cells exhibit signs of metabolic exhaustion, including reduced energy production and impaired functional capacity (9, 10).

While these data have deepened our understanding of systemic immune dysfunction, most of them stem from studies on peripheral blood mononuclear cells (PBMCs). In contrast, the peritoneal compartment remains much less explored – despite growing recognition that it constitutes a distinct immunological niche in cirrhosis (11). Immune cells within ascitic fluid are exposed to a unique microenvironment, shaped by persistent local inflammation and microbial signals, which may drive compartment-specific immune responses and metabolic adaptations in the disease course of cirrhosis.

Immune cell subsets are known to engage glycolysis and oxidative phosphorylation (OXPHOS) to markedly different extents, and these differences have been linked to distinct functional capacities across cell types (12). Monocytes and B cells, for instance, have been found to express particularly high levels of glucose uptake- and OXPHOS-related machinery even at rest (12).

NK cells have been shown to upregulate both glycolysis and OXPHOS upon activation, with glycolysis specifically required for cytotoxic effector function (13). Among monocytes, the inflammatory CD16^+^ subset has been found to be more glycolytic than classical CD16^-^ monocytes (12),and lipopolysaccharide-driven activation via Toll-like receptor 4 further shifts monocytes toward glycolysis while reducing OXPHOS (14).

T cell metabolism has been found to be particularly dynamic and context-dependent: resting memory CD4^+^ T cells have been shown to sustain higher basal OXPHOS than naive T cells, alongside greater glycolytic capacity and enzyme expression, though basal glycolytic flux itself does not differ significantly between subsets at rest (15). T cell receptor engagement has been shown to drive a broad, coordinated upshift across both glycolytic and oxidative programs (12, 15), and disrupting the oxidative arm of this early response, via STAT5 inhibition or glutamine withdrawal, has been found to abolish IL-2 production, indicating that oxidative metabolism is required for T cell activation (15).

Given the specialized immune role and microenvironment of the peritoneal cavity, we hypothesized that mononuclear cells isolated from ascites exhibit distinct phenotypic and metabolic characteristics compared to their counterparts in peripheral blood. Therefore, we focused on the analysis of immunometabolism from matched ascites and blood samples from patients with decompensated cirrhosis and how changes therein might impact patients' outcome.

## 2 Patients and methods

### 2.1 Study design and data collection

Between August 2023 and April 2024, ascites and corresponding blood samples were collected from 21 patients with decompensated cirrhosis without SBP and five patients with SBP at Charité Universitätsmedizin Berlin. Peripheral blood was additionally collected from a total of 18 healthy laboratory or clinical staff members with no liver disease or other relevant medical conditions. Diagnosis of liver cirrhosis was based on liver histology or on clear evidence of portal hypertension by clinical, laboratory, and radiological findings. SBP was diagnosed based on elevated polymorphonuclear cell count (PMN) of above 250/µl (16).

Patients with HCC, immunosuppressive therapy, or non-cirrhotic ascites were excluded.

Clinical and routine laboratory data were assessed, including MELD score, liver enzymes, coagulation, renal, hematologic, and inflammatory parameters. During a 1-year follow-up period, clinical outcome data like survival, transplantation, incidence of SBP and decompensation events were retrospectively obtained through review of medical records after completion of each patient’s follow-up period.

The study was approved by the local ethics committee (Ethics-No EA2/018/23, EA2/065/21), and all patients provided written informed consent.

### 2.2 Isolation of mononuclear cells

Up to 1 liter of ascitic fluid and 20ml peripheral blood were collected and processed without refrigeration within 3 hours of collection. Cells were centrifuged at 400g, and mononuclear cells were isolated by density gradient centrifugation using Ficoll Hypaque and magnetic cell separation (MACS).

### 2.3 Spectral flow cytometry

For Spectral flow cytometry whole leukocytes of ascites and blood were used. Experiment was performed using a 3-laser Cytek Aurora system. All samples were stained for surface markers using a 29-color antibody panel (**Supplementary Table 1**). Spectral unmixing and compensation were performed using SpectroFlo software (Cytek Biosciences) and data were analyzed using FCS Express 7 (*De Novo* Software Version 7.18.0015) for manual gating and FlowJo for UMAP analysis.

### 2.4 Seahorse XF metabolic analysis

For Seahorse analysis only mononuclear cells of ascites and blood were used. 200,000 mononuclear cells per well were resuspended in XF assay media and seeded on a 96-well plate. After injection of the electron transport chain inhibitors (oligomycin, FCCP, rotenone, and antimycin A), Oxygen consumption rate (OCR) and extracellular acidification rate (ECAR) were measured using the Seahorse XF analyzer (Agilent).

### 2.5 Lactate measurement

Lactate concentrations in plasma and ascites fluid were quantified using a colorimetric lactate assay kit (Biorbyt, Cat. No. orb1173247) according to the manufacturer’s instructions, with samples diluted 1:8 to fall within the linear range of the assay. Absorbance was measured at 450 nm and concentrations calculated by interpolation against a standard curve. Full assay details are provided in the **Supplementary Methods**.

### 2.6 Statistical analysis

Data are presented as mean ± SEM. Normality of continuous variables was assessed using the Shapiro–Wilk test (**Supplementary Table 2**). As several variables, including glycolytic potential and select immune cell subset frequencies, deviated significantly from a normal distribution, nonparametric statistical tests were applied consistently throughout the study. Statistical significance was determined using appropriate tests (as noted in figure legends), with p ≤ 0.05 considered significant. GraphPad Prism (Software Version 9.3.0) was used for statistical analysis and graph preparation. Normality testing was performed using IBM SPSS Statistics (Version 32). Figures were created using Biorender (https://biorender.com/).

Full methods and additional details are provided in the **Supplementary Materials**.

## 3 Results

### 3.1 Baseline characteristics

21 patients without and 5 patients with SBP were included in the study. The majority of patients presented with alcohol-related liver disease (ALD), followed by mixed alcohol and viral etiologies. Patients with SBP had numerically higher MELD scores (median 28.0, IQR 13.0–33.5) than those without SBP (median 15.0, IQR 10.0–19.5; p= 0.08). CRP levels were significantly higher in SBP patients (median 88.8 mg/l, IQR 45.2-108) compared to non-SBP patients (median 18.6 mg/l, IQR 6.85–33.6, p=0.013). During the one-year follow-up, mortality was 80% in patients with SBP and 36.8% in those without SBP (p = 0.142).

Among patients without SBP, seven patients died during the follow-up period, while two underwent liver transplantation. Three patients had a documented SBP episode and one patient experienced ACLF within 14 days prior to inclusion. During follow-up, seven patients developed SBP, and four experienced ACLF. The most frequent complications in patients without SBP at the time of inclusion were hydropic decompensation events (83.3%), bloodstream infections (66.7%), and hepatorenal syndrome (44.4%).

A complete overview of patient characteristics is provided in **Table 1**.

**Table 1:** SBP, Spontaneous Bacterial Peritonitis; AD, Acute Decompensation; MASLD Metabolic Dysfunction-Associated Steatotic Liver Disease; MELD, Model for End-Stage Liver Disease; AF, Ascitic Fluid; PMN, Polymorphonuclear cells; INR, International Normalized Ratio; AST, Aspartate Aminotransferase; ALT, Alanine Transaminase; TIPS, Transjugular Intrahepatic Portosystemic Shunt; ACLF, Acute On Chronic Liver Failure.

Characteristic	N^a^	AD without SBP (N=21)^b^	N^a^	AD with SBP (N=5)^b^	P value^c^
**Age**	21	57.0 (43.0, 62.5)	5	53.0 (43.0, 63.5)	0.845
**Sex**	21		5		
**Male**		13 (61.9%)		3 (60.0%)	
**Female**		8 (38.1%)		2 (40.0%)	
**Etiology**	21		5		
**Alcohol**		9 (42.9%)		1 (20.0%)	
**Viral**		3 (14.3%)		1 (20.0%)	
**Alcohol + viral**		2 (9.5%)		2 (40.0%)	
**MASLD**		2 (9.5%)		0 (0%)	
**Other**		5 (23.8%)		1 (20.0%)	
**MELD score**	21	15.0 (10.0, 19.5)	5	28.0 (13.0, 33.5)	0.0838
**Child-Pugh C**	18	6 (33.3%)	5	3 (60.0%)	0.343
**Child-Pugh score**	18	9.00 (7.75, 11.0)	5	10.0 (8.0, 13.5)	0.272
**Blood leukocytes, /nl**	20	7.07 (4.79, 9.48)	5	8.59 (4.74, 10.9)	0.76
**AF leukocytes, /nl**	20	0.21 (0.15, 0.27)	5	2.33 (1.05, 2.52)	<0.001
**AF PMN, /nl**	20	0.03 (0.02, 0.08)	5	1.56 (0.35, 1.99)	<0.001
**AF protein, g/l**	20	12.4 (9.13, 16.8)	5	15.8 (15.5, 17.4)	0.292
**Total serum bilirubin, mg/dl**	21	1.74 (1.20, 5.59)	5	5.13 (2.35, 33.7)	0.134
**Serum albumin, g/l**	18	30.5 (27.4, 37.4)	5	37.9 (28.4, 40.1)	0.682
**INR**	21	1.24 (1.15, 1.48)	5	1.58 (1.40, 1.60)	0.104
**Creatinine, mg/dl**	21	1.00 (0.780, 1.34)	5	1.40 (1.27, 1.40)	0.118
**AST, U/l**	21	62.0 (38.0, 81.0)	5	143 (41.0, 185)	0.329
**ALT, U/l**	21	33.0 (20.0, 39.0)	5	64.0 (18.0, 68.0)	0.515
**Serum sodium, mmol/l**	21	135 (133, 138)	5	139 (138, 142)	0.109
**CRP, mg/l**	20	18.6 (6.85, 33.6)	5	88.8 (45.2, 108)	0.0131
**Prior Albumin treatment^d^**	21	13 (61.9%)	5	4 (80.0%)	0.628
**Antibiotic exposure** ^d^	21	12 (57.1%)	5	3 (60.0%)	1
**Prior culture positive infection** ^d^	21	5 (23.8%)	5	2 (40.0%)	0.588
**TIPS at inclusion**	21	3 (14.3%)	5	2 (40.0%)	0.236
**use of non-selective β-blockers**	21	7 (33.3%)	5	0 (0.0%)	0.278
**Prior SBP**	21	3 (14.3%)	5	2 (40.0%)	0.236
**Prior ACLF**	21	1 (4.8%)	5	1 (20.0%)	0.354
**Clinical Outcomes (1 year)** ^e^					
**Death**	19	7 (36.8%)	5	4 (80.0%)	0.142
**SBP**	18	7 (38.9%)	5	1 (20.0%)	0.631
**ACLF**	18	4 (22.2%)	5	1 (20.0%)	1
**Liver transplant**	18	2 (11.1%)	5	0 (0.0%)	1
**hydropic decompensation**	18	15 (83.3%)	5	5 (100.0%)	1
**blood stream infection**	18	12 (66.7%)	5	3 (60.0%)	1
**hepatic encephalopathy**	18	6 (33.3%)	5	1 (20.0%)	1
**variceal bleeding**	18	1 (5.6%)	5	1 (20.0%)	0.395
**hepatorenal syndrome**	18	8 (44.4%)	5	3 (60.0%)	0.64

^a^ number of patients with available data for each parameter.

^b^ Data are presented as medians (interquartile range) or n (%), unless otherwise specified.

^c^ P values were calculated using the Mann-Whitney U test or Fisher’s exact test, as appropriate.

^d^ within 14 days prior to inclusion.

^e^ Patients lost to follow-up were excluded; events prior to death or transplantation were included if available.

#### 3.1.1 T cells are the predominant cell subset in mononuclear cells in both ascites and blood

Given the heterogeneity of mononuclear cells (MNCs) and their potentially distinct metabolic profiles, we first defined the cellular composition in each compartment to assess whether compositional differences could confound metabolic comparisons. To this end, we performed flow cytometry to characterize MNCs (excluding neutrophils) in both ascites and blood. As an initial overview, we applied UMAP analysis to visualize the MNC composition, which highlighted distinct clusters of major immune subsets and allowed discrimination of their compartmental origin (**Figure 1A**). To complement this qualitative overview, we performed conventional manual gating to obtain precise quantitative information on the frequency of the individual subsets (**Figure 1B**).

**Figure 1:**
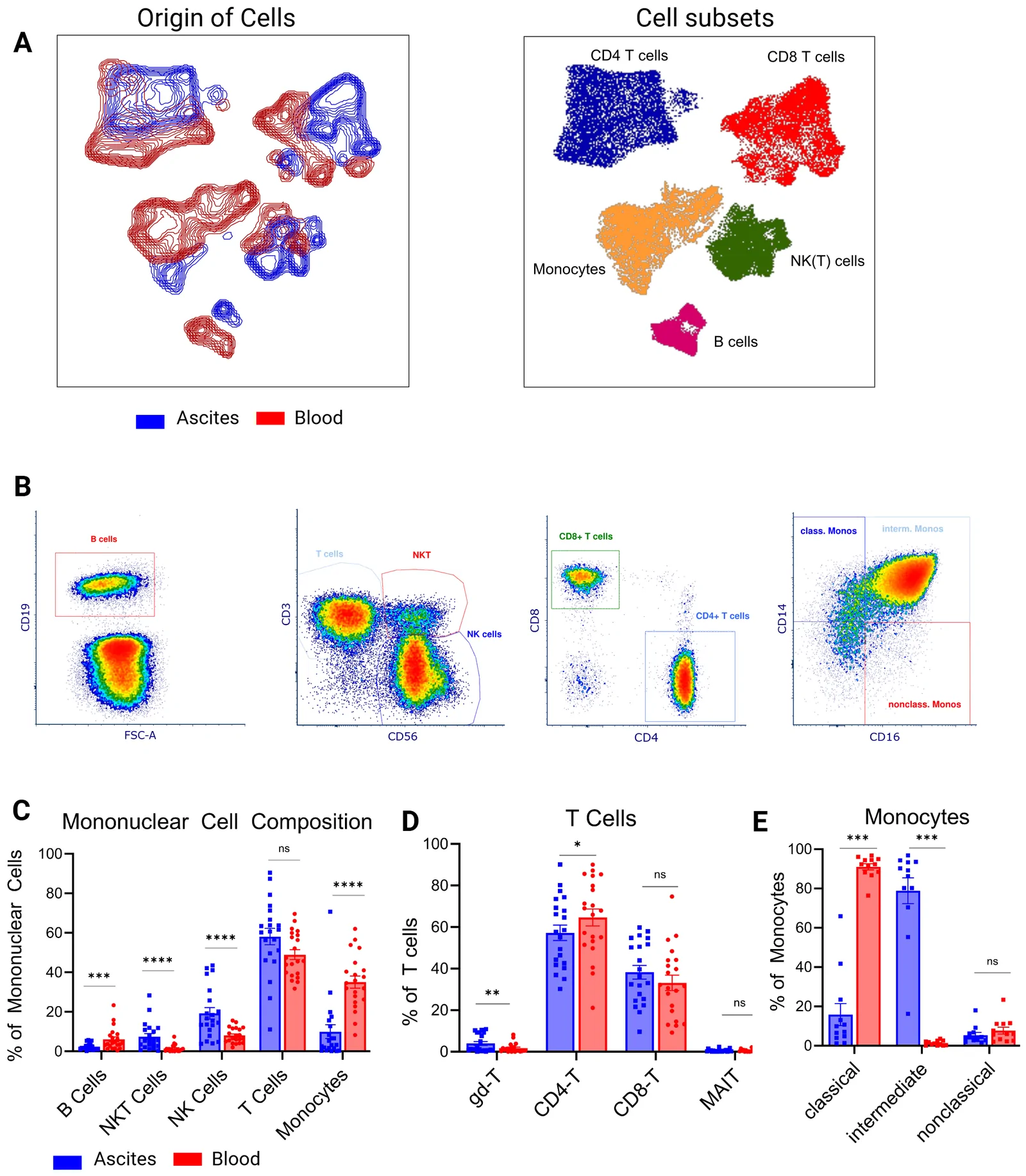
T cells constitute the majority of mononuclear cells in both ascites and blood, with distinct compositional differences in less prevalent cell subsets. **(A)** UMAP of mononuclear cells from matched ascites and blood samples (n = 21). Each sample was downsampled to 20,000 mononuclear cells before concatenation for dimensionality reduction. Left: cell distribution by sample origin (ascites vs. blood). Right: visualization of major immune cell subsets based on lineage marker expression. **(B)** Representative flow cytometry plots illustrating the manual gating strategy for identifying T cells, B cells, NK cells, NKT cells, monocytes, from a single ascites sample. The full gating strategy and use of FMO controls are provided in **Supplementary Figures 6 and 7**. **(C)** Composition of the mononuclear cell compartment in ascites and blood from patients without SBP following exclusion of neutrophils, reflecting the cell population used for subsequent metabolic analyses (n = 21). **(D)** Composition of T cell subpopulations in ascites and blood from patients without SBP (n=21). **(E)** Composition of monocyte subpopulations in ascites and blood from patients without SBP. Analysis was limited to n = 12 ascites samples in which monocytes comprised ≥2.5% of live CD45^+^ cells to ensure reliable subset characterization. In blood, all 21 samples were included. A corresponding analysis including all ascites samples irrespective of monocyte abundance is shown in **Supplementary Figure 4** and yielded same results. Wilcoxon matched-pairs test or Mann-Whitney-Test when appropriate was used. ****p < 0.0001; ***p < 0.001; **p < 0.01; *p < 0.05; ns, not significant. Absolute cell numbers are provided in **Supplementary Figure 10**.

In both ascites and blood, T cells represented the most abundant mononuclear subset, accounting for 58.2% and 49% of total MNCs respectively (**Figure 1C**). While their frequency was higher in ascites, the difference was not statistically significant (p = 0.08).

Among the less abundant MNC populations, NK cells were relatively enriched in ascites (19.3% vs. 8.1%, p < 0.001). In contrast, monocytes were more frequent in blood (35.1% vs. 10%, p < 0.0001). NKT cells and B cells were low in both compartments, with B cells slightly more enriched in blood (6.1% vs 2.4%, p < 0.001) and NKT cells enriched in ascites (7.5% vs. 1.5%, p < 0.0001). These compositional differences in the minor subsets are notable, but overall T cells remained the predominant population in both compartments.

Within the T cell compartment, CD4^+^ T cells represented the predominant subset in both ascites and blood, accounting for 57.3% and 64.6% of total T cells, respectively (**Figure 1D**). Although the difference was modest, CD4^+^ T cell frequencies were significantly higher in blood (p = 0.026). CD8^+^ T cells formed the second-largest T cell population, comprising 38.3% in ascites and 33.2% in blood (p= 0.07).

Less frequent T cell subsets were also examined. γδ T cells were present at low levels but showed a notable enrichment in ascites compared to blood (4% vs 1.7%, p< 0.01). Mucosal-associated invariant T cells (MAIT), also displayed very low frequencies across compartments with no significant difference (0.5% vs 0.5%, p=0.70).

In blood, classical monocytes (CD14^+^CD16^-^) were the dominant monocyte subset (**Figure 1E**), comprising the vast majority of total monocytes (92.2%). In ascites, classical monocytes were significantly less abundant (15.8%, p < 0.001). Interestingly, the predominant monocyte subset in ascites were CD14^+^CD16^+^ intermediate monocytes (78.8%), whereas this cell subset was almost completely absent in blood (1.3%, p < 0.001). Nonclassical monocytes (CD14^-^CD16^+^) were present at low frequencies in both compartments (5.4% vs. 7.7%, p = 0.2), with no significant difference observed across compartments. These differences highlight the presence of a distinct monocyte landscape within the peritoneal compartment.

Taken together, these findings reinforce the observation that T cells, particularly CD4^+^ T helper cells, dominate the mononuclear cell populations in both compartments. The composition of monocyte subsets on the other hand revealed important compartmental differences between ascites and blood with enrichment of intermediate monocytes in ascites and classical monocytes in blood.

#### 3.1.2 Comparison of cirrhotic blood composition with healthy controls

The cirrhotic blood samples used for the paired blood–ascites analyses were additionally compared with healthy donor blood. Most immune subsets were broadly similar between groups; however, in healthy blood there were higher proportions of B cells (10.0% vs. 4.3% of mononuclear cells, *p* < 0.01) and T cells (62.2% vs. 45.8%, *p* < 0.001), whereas cirrhotic blood contained a larger monocyte fraction (34.6% vs. 12.5%, *p* < 0.0001). MAIT cells were also markedly reduced in cirrhosis (0.2% vs. 2.0% of T cells, *p* < 0.0001). These data are shown in **Supplementary Figure 1**.

### 3.2 Peritoneal mononuclear cells are in a metabolically more active state than their counterparts from blood and show features of metabolic impairment

We next assessed the metabolic state of matched peritoneal MNCs and PBMCs from patients with cirrhosis without SBP using the Seahorse XF Analyzer (**Figure 2A**).

**Figure 2:**
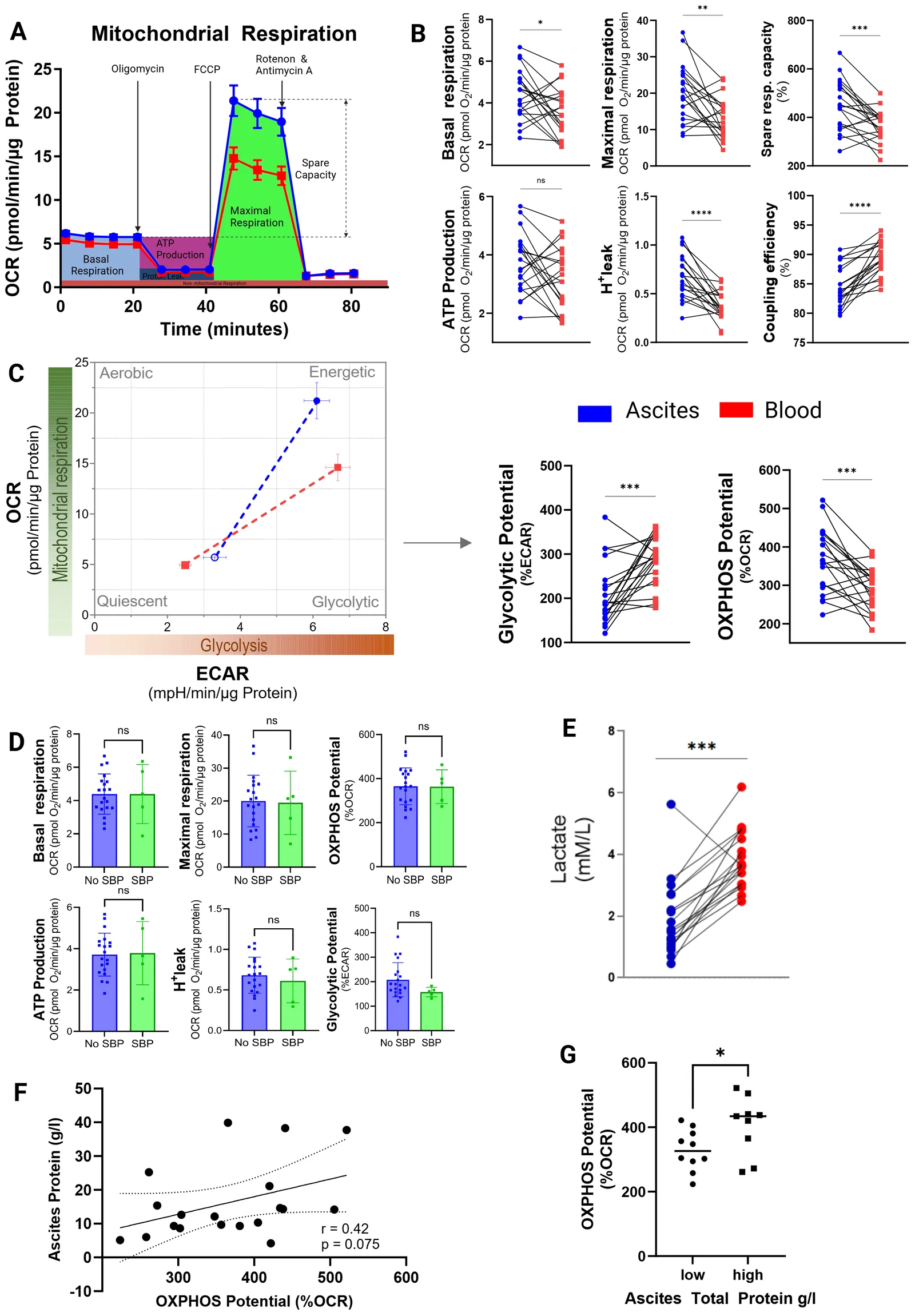
Peritoneal mononuclear cells exhibit increased oxidative respiration under basal and maximal stress conditions while showing a reduced coupling efficiency of the ETC and lower reliance on glycolysis compared to their blood counterparts. **(A)** Seahorse test principle and Mitochondrial stress test profile of matched ascites and blood samples (n=20), showing the Oxygen Consumption rates (OCR) after sequential addition of electron transport chain (ETC) inhibitors Oligomycin, FCCP, and Antimycin A &Rotenone for ascites and blood. From the original 21 matched pairs used for flow cytometry, one pair was excluded for Seahorse analysis due to a technical measurement failure. **(B)** Paired dot plots showing mitochondrial parameters—including basal respiration, maximal respiration, spare respiratory capacity, ATP production, proton leak (H^+^ leak), and coupling efficiency in mononuclear cells from paired ascites and blood samples of patients without SBP (n = 20). **(C)** XF Cell Energy Phenotype plot displaying mitochondrial respiration (OCR) and glycolysis (ECAR) of matched ascites and blood mononuclear cell samples from patients without SBP (n=20). Paired dot plots on the right illustrate the computed metabolic potentials for OXPHOS as % of OCR and for Glycolysis as % of extracellular consumption rate (ECAR), as calculated from the respective stressed versus baseline conditions. **(D)** Comparison of mitochondrial parameters—including basal respiration, maximal respiration, OXPHOS potential, ATP production, proton leak (H^+^ leak), and glycolytic potential in ascites-derived mononuclear cells from patients with (n = 5) and without SBP (n = 20). **(E)** Paired dot plot showing lactate concentrations in matched ascites and blood samples from patients without SBP (n = 18). **(F)** Scatter plot showing Spearman correlation between total protein concentration in ascites and OXPHOS potential in ascites-derived mononuclear cells (n=19). **(G)** OXPHOS potential stratified by ascites total protein levels. Patients were divided into low (n = 10) and high (n = 9) protein groups using a median cutoff of 12.6 g/L. ilcoxon matched-pairs test or Mann-Whitney-U Test was used when appropriate. ***p < 0.001; **p < 0.01; *p < 0.05; ns, not significant.

Peritoneal MNCs showed a higher respiration both at baseline levels (4.4 vs 3.6 pmol O_2_/min/µg protein, p = 0.033) and at maximal respiration (20 vs 13.3 pmol O_2_/min/µg protein, p=0.004) than counterparts from blood, resulting in a higher spare respiratory capacity (449.6% vs 364.5%, p=0.0009), an indicator of the capability of cells to respond to an energetic demand (**Figure 2B**). While ATP-linked respiration showed no differences between both groups (3.7 and 3.2 pmol O_2_/min/µg protein, p= 0.12), proton leak, an indicator of inefficiency of the electron transport chain in the mitochondria, was highly increased in ascites (0.7 vs 0.4 pmol O_2_/min/µg protein, p < 0.0001) resulting in a reduced coupling efficiency in ascites (84.5% vs 89.3%, p < 0.0001) (**Figure 2B**).

By comparing the stressed and baseline oxygen consumption rates or respective extracellular acidification rates, we evaluated the metabolic potential for OXPHOS and glycolysis, reflecting the capacity of mononuclear cells to increase their energy demand under maximal stress conditions. Consistent with Seahorse OXPHOS parameters, peritoneal MNCs showed a higher OXPHOS potential than MNCs derived from blood (365.9% vs. 290.9%, p < 0.001) (**Figure 2C**). Interestingly, glycolytic potential was lower in peritoneal MNCs than in blood (208.0% vs. 286.8%, p < 0.001), indicating differences in preferred energy utilization pathways under stressed conditions (**Figure 2C**). Baseline ECAR (ascites: 3.3 vs. blood: 2.5 mpH/min/μg Protein, p= 0.14) and OCR rates (5.7 vs. 4.9 pmol O_2_/min/µg protein, p=0.097) showed no significant differences and were at a comparable level between ascites and blood (**Figure 2C**), while stressed OCR in ascites was higher than in blood (21.2 vs. 14.6 pmol O_2_/min/µg protein, p< 0.01). Stressed ECAR rates showed no significant difference between both groups (6.1 vs. 6.7, mpH/min/μg Protein, p=0.14).

#### 3.2.1 Metabolic comparison of cirrhotic blood immune cells with healthy controls

The cirrhotic blood samples used for the paired ascites–blood analyses were additionally compared with peripheral blood from healthy donors using identical Seahorse assays. Cirrhotic blood mononuclear cells showed a significantly reduced glycolytic potential compared with healthy controls (291.9% vs. 345.0%, *p* < 0.001), while all other metabolic parameters did not differ between groups (**Supplementary Figure 2**).

#### 3.2.2 No metabolic differences between peritoneal immune cells during SBP

To assess whether SBP alters the metabolic profile of peritoneal immune cells, we compared Seahorse-derived parameters between ascites-derived mononuclear cells from patients with and without SBP. There were no significant differences across any of the assessed metabolic parameters (**Figure 2D**). Basal respiration, maximal respiration, ATP production, proton leak, coupling efficiency and OXPHOS potential were comparable between groups. Although glycolytic activity tended to be higher in SBP-negative patients, this difference did not reach statistical significance.

To further validate the reduced glycolytic potential observed in peritoneal mononuclear cells, we directly measured lactate concentrations in matched ascites and blood samples. Ascitic lactate concentrations were significantly lower than in matched blood samples (1.9 vs. 3.8 mM/L, p<0.001; Wilcoxon matched-pairs test) (**Figure 2E**).

#### 3.2.3 Low OXPHOS potential is associated with decreased protein concentrations in ascites

We next investigated whether Seahorse-derived metabolic features of ascites-derived mononuclear cells were associated with clinical parameters reflecting disease severity and risk factors of complications. A positive correlation was observed between OXPHOS potential and ascitic protein concentration, although this did not reach statistical significance (r= 0.42, p = 0.075) (**Figure 2F**). To further explore this trend, samples were stratified based on the median ascitic protein concentration (12.6 g/l) into low and high protein groups. The OXPHOS potential was reduced in the low ascites protein group compared to the high protein concentration group (329.4 vs. 406.6, p = 0.044) (**Figure 2G**).

We did not observe correlations of Seahorse parameters with the MELD score, CRP levels or ascites mononuclear cell count. Specifically, proton leak was not correlated with MELD score (r = -0.104, p=0.672). (**Supplementary Figure 3**)

### 3.3 Reduced OXPHOS potential in peritoneal immune cells is associated with increased SBP development and one-year mortality

To assess the clinical relevance of mitochondrial function in peritoneal immune cells, we stratified patients based on ascites OXPHOS potential. Patients (n = 20) were divided into OXPHOS-high and OXPHOS-low groups according to the median value (364.5%). In all Kaplan–Meier analyses, patients who underwent liver transplantation (n = 2) or were lost to follow-up before 365 days (n = 2) were censored at the respective date.

Patients with low OXPHOS potential showed a significantly higher cumulative incidence of spontaneous bacterial peritonitis (SBP) within one year (p = 0.016) (**Figure 3A**). Among the seven patients who developed SBP during follow-up, the median time to onset was 44 days (range, 4–228 days). Baseline SBP prophylaxis (with Rifaximin or Norfloxacin) at inclusion, ascites total protein concentration, and MELD score did not differ significantly between patients who did and did not develop SBP within one year (**Supplementary Table 3**); however, given the small sample size, these comparisons are underpowered to exclude confounding.

**Figure 3:**
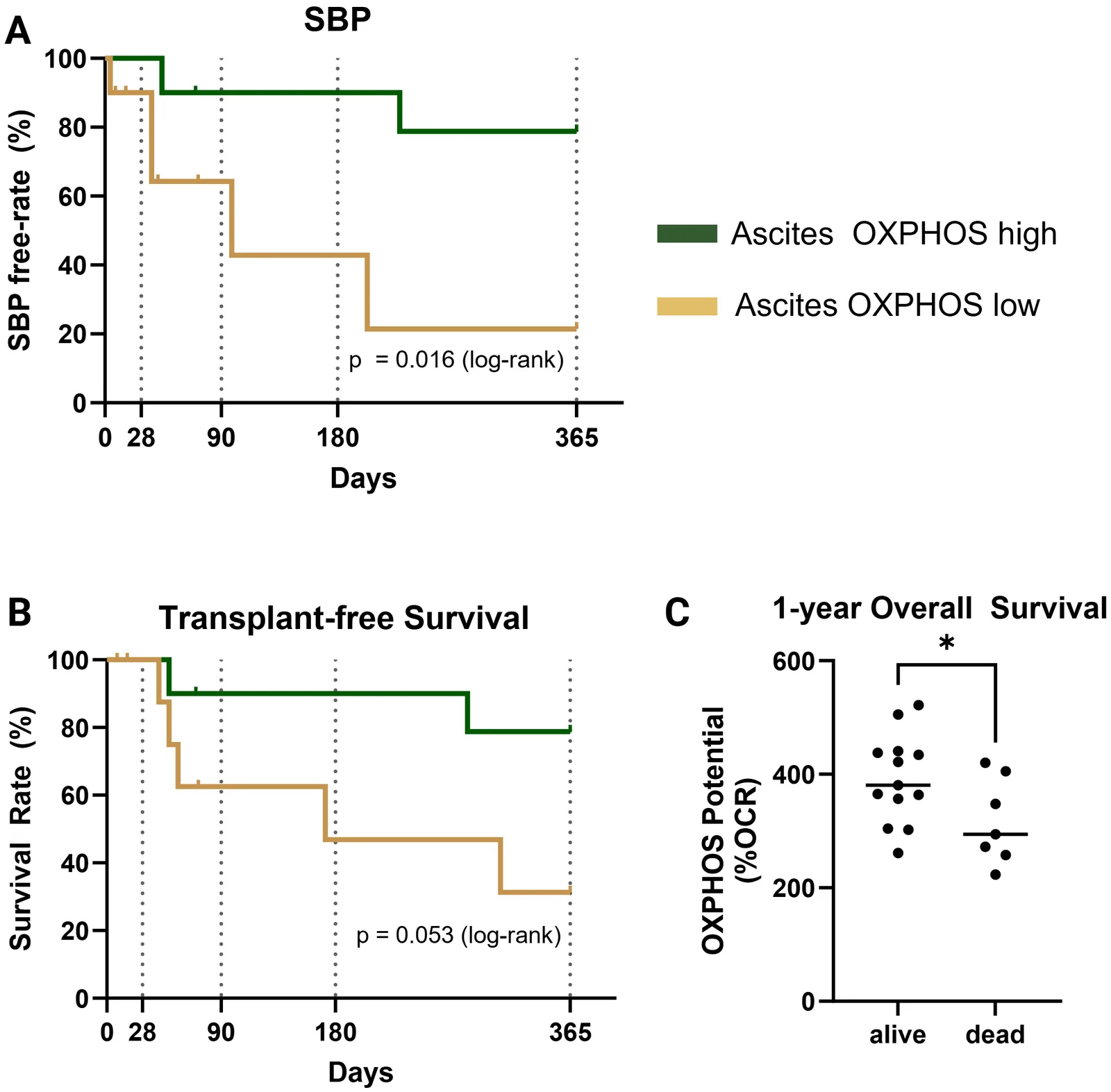
Reduced OXPHOS Potential in Peritoneal immune cells is associated with increased SBP development and one-year mortality. **(A)** Kaplan–Meier analysis showing the cumulative incidence of SBP within one year, stratified by ascites OXPHOS potential. Patients (n = 20) were divided into high and low OXPHOS groups based on the median OXPHOS potential (364.5%). Two patients who underwent liver transplantation and two patients lost to follow-up before 365 days were censored at the respective date. Log-rank p value is indicated. **(B)** Kaplan–Meier one-year survival analysis stratified by ascites OXPHOS potential. Patients (n = 20) were equally divided into high and low OXPHOS groups based on the median OXPHOS potential (364.5%). Two patients who underwent liver transplantation and two patients lost to follow-up before 365 days were censored at the respective date. Log-rank p value is indicated. **(C)** Comparison of OXPHOS potential between patients who survived at 1 year (including transplanted patients, n = 13) and those who died within 1 year (n = 7; overall survival). Mann–Whitney test was performed; *indicates p < 0.05.

In the one-year survival analysis, a trend toward reduced survival was observed in the OXPHOS-low group, which narrowly missed statistical significance (p = 0.053) (**Figure 3B**). When overall survival was analyzed, including transplanted patients, OXPHOS potential was significantly higher in patients who survived compared with those who died within one year (392.2% vs. 317.3%, p = 0.037) (**Figure 3C**).

#### 3.3.1 CD4^+^ T cells in ascites primarily exhibit an effector phenotype with elevated PD-1 expression which is associated with reduced OXPHOS potential

Having identified distinct metabolic features in both compartments, we next examined whether these were associated with functional changes in CD4^+^ and CD8^+^ T cell subsets. We assessed key surface markers of activation, differentiation, and exhaustion, including CD45RA, CD45RO, CD25, CD27, and PD-1 (**Figure 4A**) on both CD4^+^ and CD8^+^ T cell subsets (**Figures 4A–C**).

**Figure 4:**
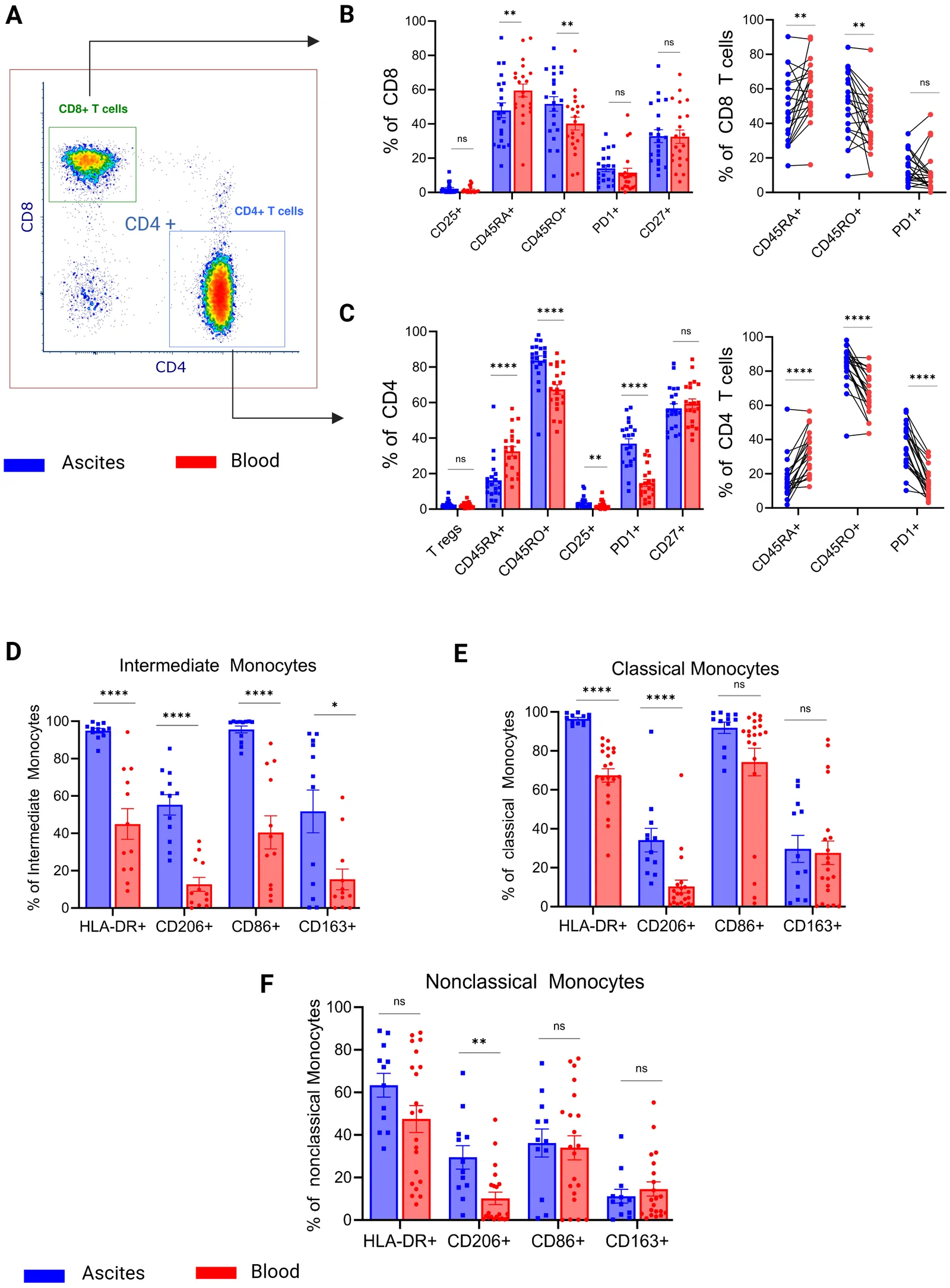
CD4+ T cells in ascites exhibit an effector/memory phenotype with elevated PD-1 expression displaying features of local immune exhaustion in the peritoneal compartment. **(A)** Representative flow cytometry plot illustrating the gating strategy for identifying CD4 and CD8 T cells from a single ascites sample. The full gating strategy is provided in **Supplementary Figure 6**. **(B)** Expression of CD25, CD45RA, CD45RO, PD-1, and CD27 on CD8^+^ T cells from matched ascites and blood samples of patients without SBP (n = 20). Data are shown as grouped bar graphs (left) and paired dot plots for CD45RA, CD45RO, and PD-1 (right). **(C)** Expression of CD25, CD45RA, CD45RO, PD-1, and CD27 on CD4+ T cells from matched ascites and blood samples of patients without SBP (n = 20). Data are shown as grouped bar graphs (left) and paired dot plots for CD45RA, CD45RO, and PD-1 (right). **(D)** Expression of HLA-DR, CD206, CD86, and CD163 on intermediate monocytes in ascites (n = 12) and blood (n = 12). Analysis was restricted to samples in which monocytes comprised ≥2.5% of total mononuclear cells in ascites, and intermediate monocytes represented ≥1% of total monocytes in blood, to ensure reliable characterization of the subset *. Man-Whitney-Test was used. **(E)** Expression of HLA-DR, CD206, CD86, and CD163 on classical monocytes in ascites (n = 12) and blood (n = 21). Analysis was restricted to samples in which monocytes comprised ≥2.5% of total mononuclear cells in ascites to ensure reliable characterization of the subset *. Man-Whitney-Test was used. **(F)** Expression of HLA-DR, CD206, CD86, and CD163 on nonclassical monocytes in ascites (n = 12) and blood (n = 21). Analysis was restricted to samples in which monocytes comprised ≥2.5% of total mononuclear cells in ascites to ensure reliable characterization of the subset *. Man-Whitney-Test was used. Wilcoxon matched-pairs test or Man-Whitney Test or Friedman Test was used when appropriate. ****p < 0.0001; **p < 0.01; *p < 0.05; ns, not significant. * A corresponding analysis including all ascites samples irrespective of monocyte abundance is shown in **Supplementary Figure 4** and yielded same results.

CD4^+^ T cells exhibited a marked shift toward the CD45RO^+^ effector/memory phenotype in both compartments. This shift was especially pronounced in ascites, where CD45RO expression was significantly higher (83.6% vs. 67.4%, p < 0.0001) and CD45RA expression significantly lower than in the matched blood samples (16.21% vs. 32.6%, p < 0.0001) (**Figure 4C**, left). Paired analysis of individual patients confirmed this difference, revealing a consistent enrichment of CD45RO^+^ CD4^+^ T cells in the peritoneal compartment. In addition to their memory phenotype, CD4^+^ T cells in ascites showed significantly elevated expression of the exhaustion marker PD-1 compared to blood (36.9% vs. 14.7%, p < 0.0001). This trend was evident in both grouped and paired analyses (**Figure 4C**, right). Median fluorescence intensity data and FMO validation for this marker are provided in the **Supplementary Results** (**Supplementary Figures 8, 9**).

Regulatory T cells (Tregs), identified as CD25^+^CD127low CD4^+^ cells, represented a minor population in both compartments and did not differ significantly in frequency between ascites and blood (2.6% vs 2.2%, p = 0.45). However, when assessing CD25 expression independently, a higher frequency of CD25^+^ CD4^+^ T cells was observed in ascites (3.9% vs. 2.3%, p < 0.01). CD27 expression, on the other hand, remained comparable between compartments (56.8% vs. 59.3%, p = 0.11).

In contrast to the CD4^+^ T cells, CD8^+^ T cells displayed a relatively stable surface marker profile. Among CD8^+^ T cells, the proportions of CD45RA^+^ and CD45RO^+^ cells in ascites were nearly equal (47.9% vs. 51.7%, p = 0.61), whereas in blood, CD45RA^+^ cells were significantly more prevalent than CD45RO^+^ cells (59.5% vs. 40.2%, p = 0.016) (**Figure 4B**, left). Paired analysis of matched samples confirmed that CD8^+^ T cells in ascites consistently showed lower CD45RA (p < 0.01) and higher CD45RO (p < 0.01) expression compared to blood, indicating a more differentiated or antigen-experienced phenotype in the peritoneal compartment (**Figure 4B**, right). Expression of the activation markers CD25 (p = 0.12) and CD27 (p=0.92), as well as the exhaustion marker PD-1 (p=0.23), showed no significant differences between ascites and blood CD8^+^ T cells.

#### 3.3.2 Peritoneal monocytes are mainly CD14+ CD16+ intermediate monocytes with highly inflammatory phenotype expressing CD206 and HLA-DR

Having established that classical monocytes were the major monocyte subset in blood and intermediate monocytes in ascites and nonclassical monocytes were present at low frequencies in both compartments, we analyzed all monocyte subsets individually for the expression of following activation and polarization-associated markers: HLA-DR, CD206, CD86, and CD163.

Monocyte frequencies in the peritoneal compartment varied considerably between patients and were very low or nearly absent in some cases. To ensure robust marker analysis, we included only samples with ≥2.5% monocytes among mononuclear cells, resulting in 12 ascitic samples. For intermediate monocytes in blood, which were largely absent, we applied an additional threshold of ≥1% among total monocytes, yielding 12 blood samples for analysis. A corresponding analysis including all ascites samples irrespective of monocyte abundance is shown in **Supplementary Figure 4** and yielded same results.

Intermediate monocytes in ascites exhibited a pro-inflammatory profile. CD206 was expressed on the majority of intermediate monocytes and showed significantly higher levels compared to blood counterparts (55.3% vs. 12.7%, p < 0.0001). HLA-DR expression was also markedly elevated in ascites, with nearly all intermediate monocytes expressing the activation marker (95.0% vs. 45.0%, p < 0.0001). Similarly, CD86 (95.6% vs. 40.5%, p < 0.0001) and CD163 (51.8% vs. 15.3%, p = 0.038) expression was found to be elevated in ascites as well (**Figure 4D**).

Classical monocytes followed a similar trend and showed increased expression of HLA-DR (96.5% vs. 67.4%, p < 0.0001) and CD206 (34.2% vs. 10.4%, p < 0.0001) in ascites, although CD86 showed increased expression of HLA-DR (96.5% vs. 67.4%, p < 0.0001), CD206 (34.2% vs. 10.4%, p < 0.0001) and CD86 (91.9% vs. 74.3%, p = 0.024) in ascites, while CD163 expression (29,7% vs. 27,6%, p =9,65) showed no difference between compartemnts (**Figure 4E**).

Nonclassical monocytes showed no significant differences in HLA-DR (ascites: 63.3% vs. blood: 47.5%, p = 0.12), CD86 (36.1% vs. 33.9%, p = 0.78), or CD163 expression (11.2% vs. 14.5%, p = 0.70) between ascites and blood (**Figure 4F**). CD206 expression was modest but significantly elevated in ascites-derived nonclassical monocytes (29.4% vs. 10.1%, p < 0.01).

Together, these results highlight that peritoneal monocytes—particularly the CD14^+^CD16^+^ intermediate subset—exhibit a highly activated surface marker profile, with consistently elevated expression of HLA-DR and CD206. These phenotypic features further distinguish the monocyte landscape in ascites from that of circulating blood.

In healthy blood, monocyte subset composition was comparable to cirrhotic blood, with classical monocytes representing the vast majority of monocytes in both groups (91.3% vs. 94.4%, *p* > 0.05). Within the classical monocyte compartment, HLA-DR expression was higher in healthy donors than in cirrhosis (84.5% vs. 66.7%, *p* < 0.01), while remaining parameters showed no significant differences between groups (**Supplementary Figure 1**).

### 3.4 High PD-1 expression on CD4^+^ T cells is associated with reduced OXPHOS potential

To explore potential links between metabolic and immunophenotypic profiles, we performed correlation analyses integrating Seahorse and flow cytometry data of 20 matched ascites samples. First, we generated a correlation matrix only including major mononuclear cell subsets — T cells, B cells, NK cells, NKT cells, and classical, intermediate, and nonclassical monocytes — and examined their relationships with Seahorse-derived metabolic parameters, including basal respiration, maximal respiration, spare respiratory capacity, ATP production, H^+^ leak, coupling efficiency, OXPHOS potential, and glycolytic potential (**Figure 5A**).

**Figure 5:**
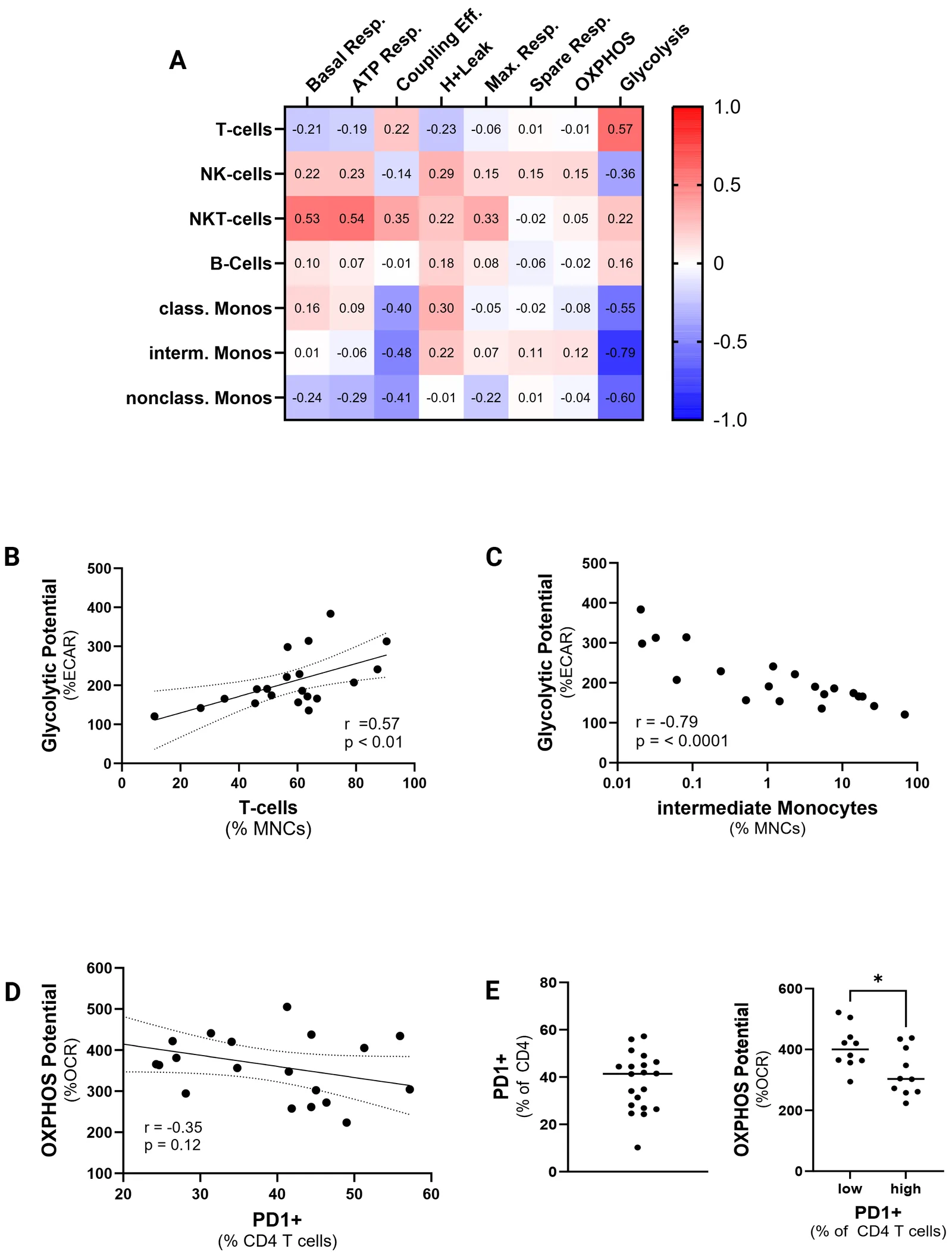
High PD-1 expression on CD4^+^ T cells is associated with reduced OXPHOS potential, supporting a link between phenotypic immune exhaustion and mitochondrial dysfunction. **(A)** Spearman correlation matrix of Seahorse metabolic parameters and mononuclear cell subset frequencies in ascites. Immune subset frequencies, expressed as percentages of mononuclear cells (MNCs), were correlated with metabolic parameters from matched ascites samples of patients without SBP (n = 20). Color scale indicates the strength and direction of correlation (red = positive, blue = negative). **(B)** Scatter plot showing Spearman correlations between glycolytic potential and relative frequency of T cells and intermediate monocytes (bottom) within the mononuclear cell compartment in ascites (n=20). **(C)** Scatter plot showing Spearman correlations between glycolytic potential and intermediate monocytes within the mononuclear cell compartment in ascites (n=20). **(D)** Scatter plot showing Spearman correlation between OXPHOS potential and CD4+ PD1 T cells within the mononuclear cell compartment in ascites (n=20). **(E)** PD-1 expression on CD4+ T cells in ascites and associated OXPHOS potential. Left: Frequencies of PD-1^+^ CD4^+^ T cells in individual ascites samples (n = 20); samples were stratified at the median into PD-1 low (n=10) and PD-1 high (n=10) groups. Right: Comparison of OXPHOS potential between the two groups. Spearman r was used to correlate among non-parametric data; Mann-Whitney-U Test was used *p < 0.05; ns, not significant.

While total T cell frequency correlated positively with glycolytic potential (r= 0.57, p = 0.009) (**Figure 5B**), intermediate monocytes (m small) frequency correlated negatively with glycolytic potential (r = -0.79, p < 0.0001) (**Figure 5C**). Correlations involving other major cell subsets and Seahorse parameters were either weak, not statistically significant, or involved minor populations with low overall abundance, and were therefore not pursued further in the analysis.

Interestingly, the percentage of PD-1^+^ CD4^+^ T cells showed a negative correlation with OXPHOS potential (r = - 0.35), although this correlation did not reach statistical significance (p = 0.12) (**Figure 5D**). Notably, we observed considerable heterogeneity in PD-1 expression levels among ascites-derived CD4^+^ T cells across patients. To further investigate this, we stratified samples into PD-1-low and PD-1-high groups based on the median PD-1 expression (41.3%) (**Figure 5E**). This analysis revealed that CD4^+^ T cells from the PD-1-low group exhibited significantly higher bulk ascites MNC OXPHOS potential compared to those from the PD-1-high group (407.1% vs. 324.6%, p = 0.036), indicating that elevated PD-1 expression is associated with reduced mitochondrial respiration.

Altogether, these findings define a distinct CD4^+^ T cell phenotype in ascites, characterized by a predominance of effector/memory cells, elevated PD-1 expression, and an association of high PD-1 levels with reduced OXPHOS potential.

## 4 Discussion

In this study, we characterized mononuclear cells in paired ascites and blood samples from patients with decompensated cirrhosis without SBP. We report that immune cells in ascites displayed a distinct phenotype, marked by an accumulation of CD4^+^ T cells with features of chronic activation and exhaustion. CD4^+^ T cells remained the dominant subset in both compartments, consistent with the expected CD4:CD8 ratio reported in the literature (17). The significant difference within the T cell compartment specifically, but not overall T cell frequency, likely reflects minor proportional shifts that, in this small cohort, differentially affected statistical significance across the two comparisons. Activated CD4^+^ T cells are intrinsically more oxidative than CD8^+^ T cells (18, 19); as CD4^+^ frequency did not differ compartmentally in a direction explaining the OXPHOS difference, activation state and local cues are more likely drivers than composition. Blood’s higher relative monocyte content, a cell type that is primarily glycolytic upon activation (14), may additionally contribute to blood’s higher glycolytic potential. These phenotypic signatures were accompanied by metabolic alterations, suggesting a close interplay between functional state and bioenergetic regulation. Indeed, studies show that T cell fate and function are critically shaped by their metabolic programming – a concept central to the field of immunometabolism (20).

Peritoneal mononuclear cells showed elevated baseline OXPHOS, yet retained the capacity to increase respiration under maximal stress, indicating a state of chronic metabolic activation in the peritoneal environment. At the same time, increased proton leak pointed to leaky mitochondria, possibly reflecting a compensatory mechanism to lower membrane potential and limit reactive oxygen species (ROS) production (21, 22). Proton leak was not correlated with MELD score, and direct markers of oxidative stress, such as ROS production, were not assessed in the current study.

Notably, the metabolic alterations we observed may have clinical implications. Patients with lower OXPHOS potential in peritoneal mononuclear cells were more likely to develop SBP. While SBP is known to result from bacterial translocation into the peritoneal cavity, this alone is not sufficient to trigger infection (23). Impaired innate and adaptive immune responses are likely to contribute to increased susceptibility (24). Upon activation, immune cells require substantial energy for effector functions, for which OXPHOS is an important source (25). Patients with low peritoneal OXPHOS potential may therefore be less able to mount an adequate immune response, increasing susceptibility to SBP.

Moreover, patients with lower ascitic fluid protein concentrations exhibited significantly reduced OXPHOS potential in peritoneal mononuclear cells. Although low ascitic protein is a known clinical risk factor for SBP (26), this association did not reach statistical significance in our cohort. Nonetheless, its parallel to the observed OXPHOS deficit is noteworthy. Previous work showed that oxidative burst and phagocytic capacity of impaired ascitic neutrophils could be partially restored by autologous plasma incubation (27), emphasizing the role of the surrounding microenvironment.

We also examined the relationship between OXPHOS potential and 1-year survival. Kaplan-Meier analysis of transplant-free survival showed a clear separation between OXPHOS groups, though not statistically significant, likely due to limited sample size. However, considering overall survival, OXPHOS activity was significantly higher in patients who survived than in those who died within one year.

These metabolic changes were accompanied by distinct changes in T cell immunophenotype. In line with a recent study by Niehaus and co-authors (28), we also observed a trend toward higher PD-1 expression on CD8^+^ T cells in ascitic fluid compared to their circulating counterparts, although this difference did not reach statistical significance in our cohort. We also found a pronounced, previously undescribed increase in PD-1 expression on CD4^+^ T cells within the ascitic compartment.

While transient PD-1 expression is a normal feature of early T cell activation, sustained and elevated expression is a hallmark of T cell exhaustion. PD-1 requires continuous antigen exposure (29), a condition that is met in the peritoneal environment of cirrhotic patients. In line with this, CD4^+^ T cells in ascitic fluid exhibited higher CD45RO expression than blood-derived counterparts, aligning with findings by Inzaugarat and co-authors, who reported increased CD45RO expression and IFN-γ production in peripheral T cells from patients with MASLD-related cirrhosis (30). Our findings extend this by showing a more pronounced immune activation phenotype in ascites, likely shaped by ongoing inflammatory stimulation.

Effector T cells have been reported to rely predominantly on glycolysis at the expense of OXPHOS during activation (31). Patsoukis and co-authors demonstrated under *in vitro* conditions that PD-1 ligation in healthy donors’ T cells can shift this balance toward increased reliance on OXPHOS (32). This aligns with the overall shift we observed toward oxidative metabolism in the peritoneal compartment.

The Seahorse analyzer indirectly measures extracellular pH as an indicator of glycolytic activity (ECAR), though this signal also reflects respiratory CO_2_-derived acidification (33). As lactate is the obligate end product of glycolysis (34), the direct measurement of lactate concentrations in matched ascites and blood provides an additional, glycolysis-specific readout. The significantly lower ascitic lactate concentrations therefore further support the assumption that peritoneal mononuclear cells rely less on glycolysis than their circulating counterparts, corroborating the reduced glycolytic potential observed by Seahorse.

Peritoneal CD4^+^ T cells with high PD-1 expression showed significantly reduced OXPHOS activity compared to PD-1-low cells, suggesting a link between immune cell phenotype and metabolic state. This finding challenges the conventional view that T cell exhaustion uniformly suppresses cellular metabolism, and may instead reflect a peritoneal-adapted exhaustion phenotype — functionally impaired but metabolically primed. Mitochondrial state is known to instruct T cell fate and function (35) and mitochondrial decline is common in exhausted T cells (36, 37), yet the directionality of this relationship has remained uncertain. Recent work by Wu and co-authors (38) demonstrated that impaired mitochondrial respiration is sufficient to induce features of exhaustion, suggesting that mitochondrial dysfunction may not only accompany but actively shape the exhausted state of local CD4^+^ T cells. Immune cells more broadly adapt their metabolism to local tissue conditions, including activation state and duration of residence (39). Within T cells, spare respiratory capacity supports function under sustained stimulation (40) and chronic CD4^+^ memory T cell activation drives a shift toward dysfunctional, oxidative-stress-associated FAO/OXPHOS reliance (41), closely paralleling the phenotype observed here. Restoring oxidative phosphorylation may represent a promising concept to improve immune cell function in cirrhosis, supported by studies showing that mitochondrial respiration in monocytes can be rescued through interventions such as mesenchymal stem cell co-culture (42) or PGC-1α activation (43), thereby improving their bacterial clearance. In a proof-of-concept experiment, we tested this directly by pharmacologically blocking PD-1 in ascites-derived mononuclear cells from a patient with decompensated cirrhosis. PD-1 blockade selectively increased oxidative respiratory capacity, including basal, ATP-linked, and maximal respiration, as well as spare respiratory capacity, while leaving glycolytic potential unchanged (**Supplementary Figure 11**), suggesting PD-1 signaling selectively constrains oxidative respiratory reserve. Given the single-patient design, this preliminary finding provides initial, disease-relevant support for PD-1 blockade as a strategy to restore mitochondrial function in exhausted peritoneal CD4^+^ T cells, warranting validation in a larger cohort.

Understanding the immunometabolic alterations requires insight into the cellular composition of the peritoneal compartment. Few studies have comprehensively characterized mononuclear subsets in ascitic fluid, with most focusing on selected populations such as NK cells, MAIT cells, or monocyte subtypes (24, 28, 44–47). Monocytes in ascites predominantly displayed a CD14^+^CD16^+^ intermediate phenotype, whereas blood-derived monocytes were mainly CD14^+^ classical cells. This intermediate subset has previously been described as pro-inflammatory (46) with higher CD206 expression in ascites, consistent with Stengel and colleagues (47), in contrast to lower CD206 levels on classical monocytes; neither total nor intermediate monocyte abundance correlated with systemic inflammatory markers such as CRP or peripheral leukocyte count.

Several limitations should be considered. One limitation is the lack of an appropriate healthy ascitic fluid control, as no ideal equivalent exists. Peritoneal fluid from patients undergoing peritoneal dialysis, used as a comparator in some studies, contains high levels of glucose and other solutes (48) that may directly affect cellular metabolism. For this reason, we did not include such samples for our Seahorse experiments.

Another limitation relates to the compartmental comparison of mononuclear cells, as these represent a heterogeneous group of cells. Although we performed flow cytometry and Seahorse assays on the same samples, allowing determination of cellular composition, measured values still reflect averaged activity across a mixed population. Thus, changes in cell metabolism cannot be directly attributed to specific subsets, allowing only for correlative analyses. Nevertheless, we detected direct associations between flow cytometric phenotypes and metabolic parameters, supporting the relevance of the observed patterns. Heterogeneity in cirrhosis etiology, age, and disease severity within our cohort may also have obscured subgroup-specific effects. The overall sample size of this cohort (n = 21) is modest; validation of the OXPHOS-SBP association in a larger, independent cohort is warranted before clinical translation. As patients were recruited at a single tertiary center, generalizability of these findings to other healthcare settings and patient populations remains to be established. Moreover, our SBP subgroup consisted of only five patients, three already receiving antibiotic therapy at sampling. This small, potentially confounded subgroup may explain the lack of observed differences in mitochondrial respiration parameters between SBP and No SBP patients. Finally, this study provides a descriptive perspective, capturing associations that suggest directions for mechanistic exploration but should not be regarded as evidence of causality.

In conclusion, this study highlights the dominant presence and functional relevance of CD4^+^ T cells in the peritoneal compartment of patients with decompensated cirrhosis, revealing profound compartment-specific differences in immune composition, activation, and metabolism. Ascites-derived CD4^+^ T cells displayed a dissociated phenotype, combining elevated oxidative phosphorylation (OXPHOS) with features of immune exhaustion; notably, high PD-1 expression was directly associated with reduced OXPHOS potential, linking these two phenomena rather than reflecting independent processes. Low peritoneal OXPHOS potential was in turn associated with increased risk of SBP development, highlighting a potential link between local immunometabolic dysfunction, immune exhaustion, and clinical outcome in decompensated cirrhosis. Further studies are needed to explore whether targeting CD4^+^ T cell exhaustion and their immunometabolism may offer new therapeutic opportunities in advanced cirrhosis.

## Data Availability

The original contributions presented in the study are included in the article/**Supplementary Material**. Further inquiries can be directed to the corresponding author.
