# Supplementary material for: Altered OXPHOS potential in ascites immune cells is linked to CD4 T-cell exhaustion and a higher risk of spontaneous bacterial peritonitis in cirrhosis
Source: Front Immunol. 2026 Oct 6;17:1853973. doi: 10.3389/fimmu.2026.1853973 (PMC13680204; doi:10.3389/fimmu.2026.1853973)
Supplement: Supplementary file 1 [file DataSheet1.docx]

**Supplementary material**

**Altered OXPHOS potential in ascites immune cells is linked to CD4 T-cell exhaustion and a higher risk of spontaneous bacterial peritonitis in cirrhosis**

Hasan Tarik Cosgun^1 2^, Pavitra Kumar^1^, Ingrid Wei Zhang^1^, Linda Hammerich^1^, Virein Sudheer^1^, Charalampos Pavlidis^1^, Juan Wang^1^, Hannah Schwarz^1^,
Frank Tacke^1^, Ali Canbay^2^, Cornelius Engelmann^1*^

^1^ Department of Hepatology & Gastroenterology, Charité – Universitätsmedizin Berlin, Campus Virchow-Klinikum and Campus Charité Mitte, 13353 Berlin, Germany

^2^ Section of Hepatology and Gastroenterology, Department of Internal Medicine,
 Knappschaft Kliniken-University Hospital Bochum, Ruhr University Bochum, Bochum,
 Germany.

*Author to whom correspondence should be addressed.

**Corresponding author:** Cornelius Engelmann;

Email: [cornelius.engelmann@charite.de](mailto:cornelius.engelmann@charite.de); Tel.: +49-30-450-553022; Fax: +49-30-450-553902; Charité Universitätsmedizin Berlin Campus-Virchow-Klinikum Augustenburger Platz 1, 13353 Berlin; Intern: Forum 4, Raum 2.0704a

**1 Methods**

**1.1 Isolation of Mononuclear cells from ascites and blood**

Up to 1 liter of ascitic fluid was collected per patient and processed without refrigeration within 3 hours of collection. Following centrifugation at 400 g for 10 minutes, the cell pellet was resuspended in PBS without calcium and magnesium PBS, and red blood cells were lysed using ammonium-chloride-potassium (ACK) buffer. Mononuclear cells were then isolated by magnetic cell separation using MACS columns (Miltenyi Biotec) following the manufacturer’s instructions. Briefly, the isolated whole leukocytes were incubated with anti-CD15 magnetic beads for 15 minutes, washed, and applied to a magnetic column. CD15⁺ neutrophils were retained within the column, and the CD15⁻ fraction containing mononuclear cells were collected, washed, and used for Seahorse Analysis

PBMCs were isolated from 20 mL of EDTA-anticoagulated blood within 3 hours of collection. Blood samples were first centrifuged at 200 × g for 10 minutes to separate plasma. The remaining cell fraction was diluted with PBS to a final volume of 20 mL and carefully layered over 13.3 mL of Ficoll-Hypaque. Samples were centrifuged at 500 × g for 25 minutes at room temperature with the brake off. PBMCs were collected from the mononuclear cell layer, followed by red blood cell lysis using ACK buffer. Cells were washed with PBS-, and if necessary, lysis and wash steps were repeated. Isolated PBMCs were used for Seahorse metabolic analysis.

**1.2 Analysis of Immunophenotype by Spectral Flow Cytometry**

To characterize the immune cell landscape in ascites and blood, spectral flow cytometry was performed using a 3-laser Cytek Aurora system. Ascites-derived whole leukocytes were isolated as described above, while blood samples were prepared by lysing 200 µL of whole blood directly in FACS tubes using BD Pharm Lyse buffer (BD Biosciences). All samples were stained for surface markers using a 29-color antibody panel (Supplementary Table 1). A fixable viability dye (Zombie NIR, BioLegend) was added for dead cell exclusion, followed by a 20-minute incubation with fluorochrome-conjugated antibodies in blocking buffer at room temperature in the dark. After staining, cells were washed twice with PBS, fixed in 2% paraformaldehyde (PFA), and stored at 4 °C for 1-3 days prior to acquisition. Precision counting beads (BioLegend) were added before acquisition for absolute cell quantification. Fluorescence-minus-one (FMO) controls were included for PD-1 (PE-Fire640) (Supplementary Fig. 8), and for HLA-DR (BV570), PD-L1 (BV650), CD163 (AF647), CD206 (PE), and TLR4 (SB436) (Supplementary Fig. 7). T cells were defined as CD3⁺CD56⁻, NK cells as CD56⁺CD3⁻, NKT cells as CD56⁺CD3⁺, B cells as CD19⁺, and neutrophils as CD66b⁺. Monocytes were classified into classical CD14⁺CD16⁻, intermediate CD14⁺CD16⁺, and nonclassical CD14⁻CD16⁺ subsets and quantified together as the total monocyte population. For the full gating strategy see Supplementary Figure 6.

Spectral unmixing and compensation were performed using SpectroFlo software (Cytek Biosciences) and data were analyzed using FCS Express 7 (De Novo Software Version 7.18.0015) for manual gating and FlowJo (V10) for UMAP analysis.

**1.3 Measurement of Oxygen Consumption and Extracellular acidification Rate using Seahorse XF Analyzer (Agilent)**

After isolation, mononuclear cells were resuspended in XF assay media (Agilent) supplemented with 10mM glucose, 1mM pyruvate and 2mM L-glutamine. 200,000 Mononuclear cells were seeded on each well of a 96-well plate with 8-16 replicate wells per sample and the seeded cell plate was incubated in an incubator at 37°C without CO_2_.

The well plate was precoated with 0.1% Poly-L-Lysine and incubated in a humidified incubator at a temperature of 37°C and a CO2 concentration of 5% for at least 1 hour to facilitate cell adhesion. For the measurement of Oxygen consumption rate (OCR; pmoles/min) and Extracellular acidification rate (ECAR; mpH/min), the culture medium was replaced with XF Calibrant solution (Agilent). In order to analyze the OCR and ECAR, specific inhibitors of the ETC including oligomycin (2 µM), FCCP (1 µM), rotenone (1 µM), and antimycin A (1 µM) were titrated and delivered through injection at designated ports A, B, and C of the Bioanalyzer. After real-time injection of the inhibitors, parameters of Oxidative Phosphorylation (OXPHOS) and Extracellular Acidification (ECAR) were measured. The resulting data were then normalized to protein concentration of whole mononuclear cell lysates to account for the total number of cells present in each well.

**1.4. Lactate Measurement**

Quantitative determination of L(+)-lactate levels in plasma and ascites fluid was performed using a commercial Lactate Colorimetric Assay Kit (Cat. No. orb1173247, Biorbyt) according to the manufacturer’s instructions. Briefly, frozen samples were thawed completely on ice and vortexed thoroughly. Due to the high endogenous concentrations of lactate in clinical samples, human plasma and ascites fluids were diluted 1:8 using the provided 1× Assay Buffer to bring the concentration within the linear detection range of the standard curve. Diluted samples and standard solutions were added in duplicate at a volume of 50 µL per well into a clear, flat-bottom 96-well microplate. Then, 50 µL of the freshly prepared Assay Reaction Mix (containing the enzyme mix and WST-8 substrate probe) was added to each well and mixed gently using a plate shaker and incubated at 37°C for 30 minutes. The absorbance was measured at 450 nm using a microplate reader. Blank-corrected absorbance values were utilized to generate the standard curve equation. The final L(+)-lactate concentrations in the plasma and ascites fluid samples were calculated by interpolating their respective OD readings against the linear regression of the standard curve. The resulting concentration values were mathematically multiplied by a dilution factor of 8 to represent the absolute concentrations in the undiluted biological samples. Data are expressed in millimoles per litre (mmol/L).

**1.5 PD-1 Blockade Experiment**

Ascites-derived mononuclear cells from one patient with decompensated cirrhosis without SBP were isolated as described above and resuspended in culture medium. Cells were incubated for 8 hours with the PD-1/PD-L1 inhibitor BMS-1 (1 μM; MedChemExpress, Cat. No. HY-19991; prepared from a 10 mM stock in DMSO) or with an equivalent volume of DMSO vehicle alone as control (final DMSO concentration: 0.1% v/v). Following incubation, cells were analyzed using the Seahorse XF Mito Stress Test as described in Section 1.3, comparing technical replicate wells of PD-1-inhibited versus control (vehicle-treated) cells from this single patient.

**1.6 Statistical Analysis**

Data are presented as mean ± standard error of the mean (SEM). Statistical significance was determined using appropriate tests (as noted in figure legends), with p ≤ 0.05 considered significant. GraphPad Prism (Software Version 9.3.0) was used for statistical analysis and graph preparation. Normality testing was performed using IBM SPSS Statistics (Version 32). Figures were created using Biorender (https://biorender.com/).

**2 Supplementary Results**

**2.1 Ascitic Protein Concentration and OXPHOS Potential: Cutoff Sensitivity**

For the comparison of OXPHOS potential according to ascitic fluid total protein concentration (Fig. 2G), patients were dichotomized at the cohort median (12.6 g/L), consistent with the approach used for other subgroup comparisons in this small exploratory cohort. This outcome-independent, pre-specified approach was chosen to avoid the risk of cutpoint-optimization bias inherent to deriving a threshold from the same outcome being tested, given our limited sample size. We additionally applied the cutoff of 10 g/L (1.0 g/dL) for ascitic fluid protein described by Runyon et al. (1986). Using this cutoff, a consistent directional trend was observed, which narrowly missed statistical significance (p=0.068), most likely reflecting reduced statistical power from the resulting unequal group sizes (n=7 vs. 12). The association between ascitic fluid protein and OXPHOS potential is therefore sensitive to the specific cutoff applied, an expected limitation given the small sample size of this cohort. This finding should be interpreted as exploratory and hypothesis-generating rather than confirmatory.

**2.2 PD-1 Blockade and Mitochondrial Respiration in Ascites-Derived Mononuclear Cells**

Given the elevated PD-1 expression observed on ascites-derived CD4⁺ T cells and its association with reduced OXPHOS potential (Fig. 5D-E), we performed a proof-of-concept experiment to test whether pharmacological PD-1 blockade could restore mitochondrial respiratory capacity. Ascites-derived mononuclear cells from one patient with decompensated cirrhosis without SBP were incubated for 8 hours with the PD-1 inhibitor BMS-1 or DMSO vehicle control, followed by Seahorse Mito Stress Test analysis (technical replicates: control, n = 13; PD-1 inhibition, n = 7; Supplementary Fig. 11). Compared to vehicle control, PD-1-inhibited cells showed significantly higher basal respiration (9.9 vs. 12.51 pmol O₂/min/µg protein, p = 0.011), ATP-linked respiration (9.035 vs. 11.35, p = 0.0085), maximal respiration (32.47 vs. 47.73, p = 0.0024), and spare respiratory capacity (22.58 vs. 35.22, p = 0.0034), resulting in a higher OXPHOS potential (239.55% vs. 298.76%). Coupling efficiency (91.43% vs. 90.88%, p = 0.55), proton (H^+^) leak (0.86 vs. 1.07, p = 0.30), and glycolytic potential (210% vs. 212%) did not differ between conditions. In the XF Cell Energy Phenotype analysis (Supplementary Fig. 11B), baseline OCR was 14.07 (SEM 0.86) in control versus 16.37 (SEM 1.30) in PD-1-inhibited cells, increasing to a stressed OCR of 33.62 (SEM 2.62) versus 48.72 (SEM 3.75), respectively. Baseline ECAR was comparable between conditions (control: 5.45, SEM 0.34; PD-1-inhibited: 5.62, SEM 0.52), as was stressed ECAR (control: 11.43, SEM 0.73; PD-1-inhibited: 11.82, SEM 1.08). This selective increase in oxidative, but not glycolytic, capacity following PD-1 blockade suggests a specific link between PD-1 signaling and constrained mitochondrial respiratory reserve in ascites-derived mononuclear cells. Given the single-patient, proof-of-concept design of this experiment, these findings should be interpreted as preliminary and hypothesis-generating.

**2.3 PD-1 Median Fluorescence Intensity and FMO Validation**

PD-1 FMO controls were consistently applied to confirm gate placement (Supplementary Fig. 8A). In the subset of samples with an available FMO control (n = 13), the difference in PD1 frequency between compartments remained highly significant (37.74% vs. 18.73%, p = 0.0002; Supplementary Fig. 8B), closely matching the result in the full cohort (36.9% vs. 14.7%, p < 0.0001; Results, Section 3.4.1), confirming that gating without an FMO control in the remaining samples did not meaningfully affect the result. To assess this in a threshold-independent manner, we compared PD-1 median fluorescence intensity (MFI) across all CD4 T cells between compartments (Supplementary Fig. 9A). MFI was numerically higher in ascites, however this difference did not reach statistical significance (2017 vs. 1657, p = 0.32). We additionally compared MFI restricted to PD1⁺ CD4⁺ T cells only (Supplementary Fig. 9B), where blood showed a slightly higher signal, again without reaching statistical significance (6449 vs. 6921, p = 0.24). Despite the clearly higher percentage of PD1⁺ cells in ascites, the absence of a significant MFI difference may be attributable to an overall higher background signal in blood, which could also account for the slightly higher PD-1 signal intensity observed on PD1⁺ cells in blood. Together, these findings support the percentage of PD1 cells, validated against FMO controls, as the appropriate primary metric for this comparison.

**2 Supplementary tables and figures**

**Supplementary Figure 1 Flow cytometric comparison of mononuclear cell subsets in healthy and cirrhotic blood**

(A) Composition of the mononuclear cell compartment in healthy (n=15) and cirrhotic blood (n=21).
(B) Frequency of γδ T cells, CD4⁺ T cells, CD8⁺ T cells, and MAIT cells among total T cells in healthy (n=15) and cirrhotic blood (n=21).
(C) Expression of CD25, CD45RA, CD45RO, PD-1, and CD27 on CD4⁺ T cells in healthy (n=15) and cirrhotic blood (n=21).
(D) Expression of CD25, CD45RA, CD45RO, PD-1, and CD27 on CD8⁺ T cells in healthy (n=15) and cirrhotic blood (n=21).
(E) Frequency of classical, intermediate, and nonclassical monocytes in healthy (n=15) and cirrhotic blood (n=21).
(F) Expression of HLA-DR, CD206, CD86, and CD163 on classical monocytes in healthy (n=15) and cirrhotic blood (n=21).

Statistical significance was determined using Mann–Whitney test (*p < 0.05, **p < 0.01, ***p < 0.001, ****p < 0.0001; ns = not significant

**Supplementary Figure 1**

**Supplementary Figure 2 Seahorse Healthy Blood vs Cirrhotic Blood comparison**

Seahorse-derived mitochondrial parameters — including basal respiration, ATP production, coupling efficiency, proton leak (H⁺ leak), maximal respiration, spare respiratory capacity, OXPHOS potential, and glycolytic potential — in mononuclear cells from healthy blood donors (n = 18) and cirrhotic patients (n = 21). Statistical significance was assessed using the Mann–Whitney test (*p < 0.05; **p < 0.01; ***p < 0.001; ns = not significant).

**Supplementary Figure 2**

**Supplementary Figure 3 Correlation plots**
Correlation plots of MELD score, CRP, and ascites mononuclear cell count with selected Seahorse parameters (basal respiration, proton leak, OXPHOS potential, and glycolytic potential) measured in ascites-derived mononuclear cells (n = 19). Correlations were assessed using Spearman’s rank correlation coefficient.

**Supplementary Figure 3**

**Supplementary Figure 4 Monocyte Plots with all samples included from ascites and blood**

A) Distribution of classical, intermediate, and nonclassical monocytes in ascites and blood (n=21).

(B–D) Expression of HLA-DR, CD206, CD86, and CD163 on intermediate (B), classical (C), and nonclassical (D) monocytes in ascites and blood. All 21 ascites samples were included regardless of monocyte abundance. Statistical significance: ****p < 0.0001, **p < 0.01, ns = not significant.

**Supplementary Figure 4**

**Supplementary Figure 5**
Comparison of total protein concentrations in ascites between patients who did and did not develop SBP during the one-year follow-up

**Supplementary Figure 5**

**Supplementary Figure 6 Gating strategy**

(A) General gating strategy of CD45^+^ Leukocytes
(B) Lymphoid populations
(C) Myeloid populations

**Supplementary Figure 6**

**Supplementary Figure 7 Use of Fluorescence minus one controls (FMO)**
Use of fluorescence minus one (FMO) controls to define gating for intermediate monocytes. Left panels show representative flow cytometry plots with the gates set for HLA-DR, CD206, and CD163. Right panels show the corresponding FMO controls for HLA-DR, CD206, and CD163, used to determine background fluorescence and validate gate placement

**Supplementary Figure 7**

**Supplementary Figure 8 PD-1 gating strategy and FMO controls**

(A) Representative flow cytometry plots from one patient illustrating PD-1 gating on CD4⁺ T cells in ascites (left) and blood (right). Top row: full-stain samples, showing the percentage of PD1⁺ CD4⁺ T cells. Bottom row: corresponding fluorescence-minus-one (FMO) controls, used to define background fluorescence and validate gate placement.

(B) PD-1 expression restricted to the subset of samples with an available FMO control (ascites, n = 13; blood, n = 13). Wilcoxon matched-pairs test; ***, p < 0.001.

**Supplementary Figure 8 PD-1 gating strategy and FMO controls**

**Supplementary Figure 9 PD-1 median fluorescence intensity in ascites and blood**

(A) Median fluorescence intensity (MFI) for PD-1 across all CD4⁺ T cells in matched ascites and blood samples (n = 21).

(B) MFI for PD-1 restricted to PD1⁺ CD4⁺ T cells only, in matched ascites and blood samples (n = 21).

Wilcoxon matched-pairs test; ns, not significant.

**Supplementary Figure 9 PD-1 median fluorescence intensity in ascites and blood**

**Supplementary Figure 10 Absolute Cell numbers of flow cytometry data shown in main article**

Absolute cell numbers (cells per mL ascites or blood) are shown for mononuclear cells (MNCs), neutrophils, T cells, CD4⁺ T cells, CD8⁺ T cells, total monocytes, intermediate, classical, and nonclassical monocytes of ascites (n=21) and blood (n=21) samples. For each cell subset, the respective marker expression is provided, showing the absolute counts of cells with specific surface markers. This figure complements the relative percentages of marker expression presented in the main article by providing absolute numbers for each immune cell population and their subsets across ascites and blood samples.

**Supplementary Figure 10 Absolute Cell numbers of flow cytometry data shown in main article**

**Supplementary Figure 11 PD-1 blockade increases oxidative respiratory capacity in ascites-derived mononuclear cells**

Ascites-derived mononuclear cells from one patient with decompensated cirrhosis without SBP were incubated for 8 hours with the PD-1 inhibitor BMS-1 or with DMSO vehicle control, followed by Seahorse Mito Stress Test analysis. Data represent technical replicates from this single patient (control, n = 13; PD-1 inhibition, n = 7) and should be interpreted as proof-of-concept.

(A) Basal respiration, ATP production, coupling efficiency, proton (H+) leak, maximal respiration, and spare respiratory capacity in control (grey) versus PD-1-inhibited (gold) mononuclear cells.

(B) XF Cell Energy Phenotype plot showing baseline and stressed oxygen consumption rate (OCR) and extracellular acidification rate (ECAR) for control and PD-1-inhibited cells.

(C) OXPHOS potential and glycolytic potential, calculated as in Figure 2, for control versus PD-1-inhibited cells.

**Supplementary Figure 11 PD-1 blockade increases oxidative respiratory capacity in ascites-derived mononuclear cells**

**Supplementary Table 1 List of fluorochrome-conjugated antibodies**

**Supplementary Table 2**

Normality testing (Shapiro–Wilk) of continuous variables used in statistical comparisons (n = 20). Normality was assessed using the Shapiro–Wilk test. Testing was restricted to the n = 20 patients without SBP with complete matched ascites–blood data (one patient excluded from Seahorse analysis due to a technical measurement failure).

| **Variable** | **Shapiro–Wilk Statistic** | **df** | **Sig. (p)** |
| --- | --- | --- | --- |
| ***Mononuclear cell composition*** | | | |
| T cells (% of MNCs) | 0.966 | 20 | 0.541 |
| NK cells (% of MNCs) | 0.889 | 20 | 0.026 |
| NKT cells (% of MNCs) | 0.847 | 20 | 0.005 |
| B cells (% of MNCs) | 0.892 | 20 | 0.029 |
| Classical monocytes (% of monocytes) | 0.872 | 20 | 0.013 |
| Intermediate monocytes (% of monocytes) | 0.591 | 20 | <0.001 |
| Nonclassical monocytes (% of monocytes) | 0.858 | 20 | 0.007 |
| CD4+ T cells (% of T cells) | 0.961 | 20 | 0.558 |
| CD8+ T cells (% of T cells) | 0.932 | 20 | 0.167 |
| ***Seahorse metabolic parameters*** | | | |
| Basal respiration | 0.975 | 20 | 0.862 |
| ATP-linked respiration | 0.979 | 20 | 0.914 |
| Coupling efficiency | 0.931 | 20 | 0.158 |
| Proton (H+) leak | 0.982 | 20 | 0.956 |
| Maximal respiration | 0.963 | 20 | 0.604 |
| Spare respiratory capacity | 0.982 | 20 | 0.952 |
| OXPHOS potential (%OCR) | 0.969 | 20 | 0.725 |
| Glycolytic potential (%ECAR) | 0.889 | 20 | 0.026 |

**Supplementary Table 3. Comparison of clinical factors between patients with and without SBP development within 365 days**

| **Variable** | **No SBP** | **SBP** | **P value** |
| --- | --- | --- | --- |
| **SBP prophylaxis at inclusion (Rifaximin or Norfloxacin)** |  |  | 0.354 |
| No, n (%) | 11 (73.3%) | 4 (26.7%) |  |
| Yes, n (%) | 3 (50.0%) | 3 (50.0%) |  |
| Ascites total protein, median (IQR), g/l | 14.25 (25.18) | 10.30 (9.40) | 0.227 |
| MELD score, median (IQR) | 16.00 (10.50) | 16.00 (7.00) | 0.592 |

P values were calculated using the Mann-Whitney U test or Fisher’s exact test, as appropriate.
